# Osteoimmunopathology in HIV/AIDS: A Translational Evidence-Based Perspective

**DOI:** 10.4061/2011/359242

**Published:** 2011-05-21

**Authors:** André Barkhordarian, Reem Ajaj, Manisha H. Ramchandani, Gary Demerjian, Riana Cayabyab, Sohrab Danaie, Nora Ghodousi, Natasha Iyer, Nicole Mahanian, Linda Phi, Amy Giroux, Ercolano Manfrini, Negoita Neagos, Muniza Siddiqui, Olivia S. Cajulis, Xenia M. C. Brant, Paul Shapshak, Francesco Chiappelli

**Affiliations:** ^1^Section of Oral Biology, Division of Oral Biology & Medicine, UCLA School of Dentistry, Los Angeles, CA 90095, USA; ^2^Section of Orthodontics, Division of Associated Clinical Specialties, UCLA, School of Dentistry, Los Angeles, CA 90095, USA; ^3^Dental Sleep Clinic (Private Practice), Glendora, CA 91741, USA; ^4^Dental Group of Sherman Oaks, Inc. (Private Practice), Sherman Oaks, CA 91403, USA; ^5^Department of Endodontics and Research and Education Center, UFMG, Belo Horizonte MG 31270-901, Brazil; ^6^Division of Infectious Disease, Department of Internal Medicine, and Psychiatry and Behavioral Medicine, Tampa General Hospital, USF College of Medicine, Tampa, FL 33606, USA

## Abstract

Infection with the human immunodeficiency virus-1 (HIV) and the resulting acquired immune deficiency syndrome (AIDS) alter not only cellular immune regulation but also the bone metabolism. Since cellular immunity and bone metabolism are intimately intertwined in the osteoimmune network, it is to be expected that bone metabolism is also affected in patients with HIV/AIDS. The concerted evidence points convincingly toward impaired activity of osteoblasts and increased activity of osteoclasts in patients with HIV/AIDS, leading to a significant increase in the prevalence of osteoporosis. Research attributes these outcomes in part at least to the ART, PI, and HAART therapies endured by these patients. We review and discuss these lines of evidence from the perspective of translational clinically relevant complex systematic reviews for comparative effectiveness analysis and evidence-based intervention on a global scale.

## 1. Osteoimmunopathology in HIV & AIDS: A Case for Fragile Bones

### 1.1. Osteoimmunology

Osteoimmunology pertains to the physiological process that involves the intimate intertwining of bone metabolism and cellular immune surveillance. It is a novel interdisciplinary research field that has converged into studies of the interplay between the immune system and bone metabolism. 

Osteoimmunopathology refers to the chronic or acute inflammatory reactions subsequent to an excessive immune response that damage bones and joints, as in osteoporosis, or to the *sequelae* of bone pathologies upon regulation of cell mediated immunity. Cytokines and other soluble immune factors modulate and regulate the maturation of osteoblasts responsible for bone formation, and the osteoclastic bone resorption [1–4]. In addition, bone is richly innervated by both autonomic and sensory neurons, which serve sensory and regulatory functions, and directly mediate bone and immune cell activities in the neuroimmune network [5]. Moreover, present understanding of the neuroendocrine-immune regulatory pathway dictates that hormones (e.g., adrenocorticotropin hormone [ACTH]; [6]; parathyroid hormone and calcitonin; [7–9]) play an important role in maintaining bone metabolism, and thus contribute to bone mineralization and mass, by directly influencing the metabolism of osteoblasts and osteoclasts.

The parathyroid hormone pathway is of particular note in this context because it appears to regulate production of the proinflammatory cytokine, interleukin (IL)-6, and of the Receptor Activator for Nuclear Factor *κ* B Ligand (RANKL) (i.e., TRANCE: Tumor Necrosis Factor- [TNF-] related activation-induced cytokine, OPGL: Osteoprotegerin Ligand, ODF: Osteoclast Differentiation Factor, TNFS11). This accomplished through osteoblasts, thereby facilitating the differentiation, activation, and survival of osteoclasts [10, 11]. That is to say, parathyroid hormone and parathyroid hormone-related protein (PTH-rP) promote bone resorption by regulating OPGL (i.e., RANKL), and *de facto* the release of calcium [12, 13]. 

Estrogen deficiency results in a net bone loss as a consequence of an increase in production of RANKL, a decrease in production of OPGL by osteoblastic lineage cells, and an enhancement of the secretion of pro-inflammatory and proresorptive cytokines by lymphocytes such as IL-1, IL-6, and TNF-*α*. OPGL and its binding to the OPGL receptor located on osteoclast precursors are essential for development and activation of osteoclasts. They are critical regulators of physiological bone remodeling as in osteoporosis. OPG receptor is another regulator protein, which binds to RANKL, blocks RANK/RANKL interaction as a result inhibiting maturation of osteoclast precursors to osteoclasts. Therefore, production of OPGL by osteoblast lineage cells and activated T cells can directly regulate osteoclastogenesis and bone remodeling [14, 15]. OPGL is among the principal causes of bone and cartilage destruction, as it binds to its OPGL receptor on preosteoclastic cells, it inhibits osteoclast maturation. In a delicate regulatory loop, the natural decoy receptor osteoprotegerin (OPG) binds to OPGL, as well as RANKL, and prevents bone loss by blocking RANK and OPGL receptor activation. OPG expression is induced by estrogen, which provides a molecular explanation of postmenopausal osteoporosis in women due to decreased levels of the hormone [16]. The bone protective effect of estrogen is mediated in large part by transforming growth factor (TGF)-*β*, which induces apoptosis in osteoclast cells [17, 18], thus closing this remarkable and finely regulated balance between bone metabolism and neuroendocrine immunoregulation.

### 1.2. Osteoporosis in Otherwise Normal Individuals

Osteoporosis develops when bone that is resorbed is not replaced by new bone. It is the result of any imbalance in bone turnover that causes an excess of osteoclast activity (bone resorption) over osteoblast activity (bone formation). 

Osteoporosis is a bones disease that leads to an increased risk of fracture. It is among the most common human bone diseases and is characterized by low bone mass or bone mineral density and loss of bone tissue: bone microarchitecture is disrupted, and the amount and variety of proteins in bone is altered. The World Health Organization (WHO) in women defines osteoporosis as bone mineral density 2.5 standard deviations below peak bone mass (20-year-old healthy female average). 

Osteoporosis is most common in women after menopause (i.e., postmenopausal osteoporosis), but may also develop in men or women of any age consequential to hormonal disorders (e.g., hyperparathyroidism) and other chronic diseases, or glucocorticoids intake (i.e., steroid- or glucocorticoids-induced osteoporosis). Given its influence in the risk of fragility fracture, osteoporosis may significantly affect life expectancy and quality of life. Osteoporosis can be prevented with lifestyle changes (e.g., exercise) and sometimes medication (e.g., dietary supplements of calcium, vitamin D, bisphosphonates). The RANKL/OPG ratio has been implicated in the development and progression of fragile bones and osteoporosis [15, 19–21].

The three main mechanisms by which osteoporosis develop include

1. inadequate peak bone mass (the skeleton develops insufficient mass and strength during growth),
2. excessive bone resorption,
3. inadequate formation of new bone during remodeling.

Interplay of these three mechanisms underlies the development of fragile bone tissue. 

### 1.3. “Fragile Bones” in HIV/AIDS

Early clinical observations were made of patients infected with the human immunodeficiency virus-1 (HIV) and exhibiting the acquired immune deficiencies syndrome (AIDS). A spectrum of pathologies was found that afflicts HIV-seropositive patients as their condition worsens to HIV/AIDS. These patients demonstrated increased susceptibility to develop weak and fragile bones, decreased bone mineralization, and increased osteoporosis [22–24]. Nevertheless, the fundamental underlying biological mechanisms remained elusive, until now, as the field of osteoimmunology has become better established.

In brief, an exhaustive Cochrane systematic review established that the clinical data taken together show that decreased bone mineral density occurs more commonly in patients with HIV than in the general population, making the group of HIV-seropositive men and women more susceptible to osteoporosis and consequent fragility fractures [25]. In fact, osteoporosis in HIV-infected persons is at least as prevalent as in postmenopausal women, despite the fact that this population is not listed in primary care guidelines as one that should be considered for screening [26]. This is perhaps due to the fact that the data still fail to clearly expose the fundamental mechanisms that underlie osteoporotic processes in HIV/AIDS. 

The role of antiretroviral therapy (ART) in the prevalence estimates of osteopenia and osteoporosis in HIV-infected patients was initially equivocal [27]. However, the clinical evidence linking HIV-associated osteoporosis directly to ART intervention appears now more clearly established [28, 29]. (Refer to pages 10, 12, and 13 for a complete perspective on PI/HAART/ART impact on osteoporosis. While there is evidence in support of both the association of PI/HAART/ART and osteoporosis, as well as the opposing views, this paper supports the clinical evidence for the direct link and intervention of PI/HAART/ART and osteoporosis. For a complete perspective, opposing articles are also cited.) By contrast, data to this date fail to demonstrate consistent interactive relationships among the observed ART drug-specific effects upon clinically relevant bone mineral density decline in these patients [28].

## 2. Osteoimmunopathology and Osteoporosis

### 2.1. Bone Metabolism

Bone metabolism consists of a complex series of finely regulated steps and events, which involve primarily the activity of bone forming osteoblasts and bone destroying osteoclasts (Bone metabolism processes are generally effectively equal throughout the axial and the appendicular skeletons, although certain specific differences exist that depend upon local epigenetic regulatory and modulatory events (e.g., forces and stress upon the teeth during mastication and the consequential forces upon the alveolar bone [30, 31]).)

Osteoblasts are immature mononucleated bone-forming cells that derive from osteoprogenitor cells as a common family branch that arises from mesenchyme. The mesenchyme is a type of loose connective tissue, derived from the three embryonic germ layers (i.e., endoderm, mesoderm, and ectoderm). In ontogenic development, the mesodermal middle layer gives rise to the skeleton and the hematopoietic system [32]. Core binding factor 1 (Cbfa1) also known as the runt-related transcription factor 2 (Runx2) and the downstream factor osterix (Osx) are critical transcription factors for lineage commitment of stem cells toward osteoblast differentiation [30, 33, 34]. Although a relatively rare event, it may be hypothesized that, given the appropriate microenvironment, osteocytes and osteoblasts can revert back to earlier stages of their development. Whether ART, PI, or HAART, or more directly HIV infection may modulate and control such a reversal is emerging as a plausible working hypothesis. 

The principal proteomic signature of osteoblasts is the production and expression of alkaline phosphatase (EC 3.1.3.1), a key dephosphorylating enzyme that contributes to the accumulation of calcium and phosphate into the vesicles generated during the formation of the matrix. Four principal isozymes of alkaline phosphatase have been recognized, and the* ALPL* gene is now known to be located on chromosome 1. When alkaline phosphatase is missing, a disorder known as hypophosphatasia arises, which manifests as hypercalcemia and skeletal fragility [35]. It remains to be tested if ART, PI, or HAART, HIV infection directly or indirectly, or the manifestation of AIDS can regulate *ALPL *gene expression.

Osteoblasts also produce bone sialoprotein (a 60–80 kDa small integrin-binding ligand and a member of SIBLING proteins, a constituent of mineralized tissues such as bone, dentin, cementum, and calcified cartilage), osteocalcin (i.e., BGLAP: bone gamma-carboxyglutamic acid-containing protein) and osteopontin (OPN) (i.e., BSP-1 or BNSP: bone sialoprotein I, ETA-1: early T-lymphocyte activation, SPP1: secreted phosphoprotein 1). OPN is produced by osteoblasts as well as variety of immune cells, including macrophages, neutrophils, dendritic cells, and T and B cells. In immune metabolism, OPN is endowed with chemotactic properties that promote cell recruitment to inflammatory sites as well as adhesion properties to several integrin receptors, which promote T-cell activation, cytokine production, and regulation of apoptosis. In brief, the SIBLING protein family, including OPN, carries complex molecular and epigenetic regulatory roles in osteogenesis and in cellular immune regulation [36–38].

Recent data, moreover, implicate a significant role of OPN in the recruitment of macrophages in the central nervous system of patients with HIV/AIDS, thus suggesting an important role in the onset and progression of neuroAIDS [39]. This epigenetic factor was overlooked in previous reviews of patients with HIV/AIDS suffering from AIDS-related neurological and neurocognitive impairments [40–42].

OPN regulates the development of distinct effector T cells, such as specifically TH1 and TH17 cells [43]. In addition, OPN plays a critical role in regulating osteoclasts, which are responsible for bone resorption. Osteoclasts are large multinucleated cells that arise and derive from the myeloid common progenitors and are essentially of a parallel lineage to that of immune monocytes/macrophages. Similar to the myeloid family, osteoclasts are endowed with phagocytic properties, share several families of plasma membrane receptors with certain immune cell populations, and function in a manner similar to their mature myeloid equivalent. Osteoclasts are found on bone surfaces in what are called Howship's lacunae, bone resorption pits that result following breakdown of the bone surface and consequential erosion of the bone by the osteoclastic enzymes, including lysosomes, organic acids, and hydrolytic enzymes. The osteoclastic layer that contacts the bone is divided into the microvillus structure, a ruffled border rich in plasma membrane folding, and a ring-like perimeter of cytoplasm, termed the clear zone, that marks the area of bone in the process of being resorbed.

Research has now established that unloading of stress, such as that brought about by teeth upon the alveolar bone, results in extensive bone remodeling. In the case of the tooth sockets, teeth can move as a result of excessive unloading stress in both vertical (“supereruption”) and horizontal direction (“drift”). Animal studies show that OPN expression is necessary for the “drift” process, but not the “super-eruption” event. The biological mechanism begins to become apparent, as it was observed that RANKL expression increased significantly in the distal periodontal ligament in the first week following unloading, and the OPN-induced involvement of the PI3K and MEK/ERK pathway was demonstrated in osteoclast activation following unloading-induced drifting of teeth [44].

It has been proposed that the lateral drift imposed upon the teeth subjected to bruxism (Bruxism refers to the events characterized by the grinding of the teeth that typically includes the clenching of the jaw.) stress strain created by the oral parafunctional habit of grinding one's teeth and the paired activity of jaw clenching may be a significant participatory causative agent to bone resorption of the involved tooth socket, leading progressively to retraction of the gum line, and exposure of the dentin inferior to the dentin-enamel juncture [45–47]. Abfraction of the exposed dentin follows, with increased tooth sensitivity [48], pain, and possible involvement of the tooth pulp. While abfraction is still regarded by some as a theoretical concept [49], the model, in and of itself suggests that it is possible and even probable that the etiology and progression of this pathological process is favored in patients with osteoporosis. This hypothesis appears to be confirmed by the epidemiology of abfractions, which has a significantly higher prevalence in aging and women. Undoubtedly, the potential role of occlusal loading in the loss of cervical tooth tissue is important in the management of occlusion and must be incorporated into a treatment plan for a patient with abfraction lesions. This also pertains to patients with HIV/AIDS [50]. Research must now be crafted to test the hypothesis that patients with HIV/AIDS are at higher risk of abfractions consequentially to their increased risk of osteoporosis, and that the underlying osteoimmune mechanism involves OPN and RANKL activation following lateral drift in patients with HIV/AIDS who grind.

Bruxism generally includes side-to-side grinding and is often accompanied by jaw clenching and can be induced by stress and anxiety during both sleep and when awake. Together, they can lead to chronic discomfort and pain in the head and neck region, as well as dentition, masticatory muscle, and facial skeletal *sequelae*. In the more severe cases, clenching and grinding can result in disorders of the temporomandibular joint, which can be exacerbated by osteoporosis by means of at least two mechanisms:

1. osteoclastic activity of the cartilaginous corona of the condyle may lead to bone-on-bone frictions that engender inflammatory reactions at the joint, and consequentially more extensive osteoclast activity and accelerated bone resorption at the temporomandibular joint, which will lead to joint disorder;
2. stress loading on the osteoporotic alveolar bone will alter occlusion and lead to impaired joint function.

It is possible and even probable that many patients with periodontal disease who have alveolar bone loss nevertheless, may retain normal occlusion. Bruxism can lead to excessive wear that may alter occlusion; however, alveolar bone loss by itself may not alter occlusion in all patients. Undoubtedly, occlusion issues are not considered in all cases to be *sine qua non* for temporomandibular joint disorders [51]. Temporomandibular joint disorders represent a complex set of issues, which can involve a range of variables (e.g., psychoemotional stress of having HIV/AIDS, which may lead to depressive symptoms) such as increased bruxism, and increased tone of the masticatory musculature, which remains in need of further study in health subjects, as well as in patients with HIV/AIDS.

Furthermore, it is important to note that the prevalence of temporomandibular joint disorders is significantly higher in young adult women than in men: generally, a 2 : 1-fold increase in prevalence is reported. Although, some reports indicate that women might be up to 5 times more prone to temporomandibular joint disorders than men, matched for age. Over 80% of the patients treated for temporomandibular joint disorders are women [52–56].

In women, the severity of symptoms is related to the age of the patients. Pain onset tends to occur after puberty, and peaks in the reproductive years, with the highest prevalence occurring in women aged 20–40, and dampens in the elderly [52–54]. The putative role of estrogen for temporomandibular joint disorders in women [53] may have multiple mechanisms, including influencing cellular events in bone metabolism (*vide supra*), as well as regulatory processes of neuralgia, myalgias, migraines, and localized or generalized pain responses [52, 56]. Hormone-replacement therapy has been reported to raise the prevalence of temporomandibular joint disorders in aging postmenopausal women, although this observation has not been confirmed [55].

At the molecular level, recent data suggest involvement of bone cell estrogen receptors, whereas the precise mechanism of the osteoprotective estrogen action remains unclear. The inhibitory action of estrogen on bone resorption appears to be mediated by the osteoclastic nuclear estrogen receptor-*α* (aka, nuclear receptor subfamily 3, group A, member 1, and NR3A1), which directs a shortened lifespan of osteoclasts [57]. Related findings indicate that estrogen and glucocorticoids modulate the functional differentiation of osteoblasts differentiation that is regulated by bone morphogenic proteins (BMP's) and TNF-*α*. BMP-2 specifically increases the binding affinity of estrogen and glucocorticoid receptors, thus amplifying these outcomes. By contrast, estrogens and glucocorticoids differentially regulate the BMP-2-induced Smad1/5/8 phosphorylation and TNF-*α* signaling pathways, which participate in the regulation of target gene expression [58].

Temporomandibular joint disorders are not observed as a routine clinical feature in patients with HIV/AIDS. In addition, osteoporosis generally is regarded as a condition with high risk of developing temporomandibular joint disorders. However, the prevalence of HIV/AIDS in women of childbearing age is fast rising worldwide. Moreover, and as noted above, the diagnosis of HIV/AIDS often triggers serious symptoms of depression, which may require treatment protocols with antidepressants, including selective serotonin reuptake inhibitors (SSRI's). Clinically, these pharmacotherapeutic modes of intervention are significantly correlated not only with increased bone fragility (clinical fragility fracture hazard rate, 2.1; CI_95_ : 1.3–3.4 (*vide infra*) [59], but also with clinically relevant increased clenching of the jaw and bruxism. It was proposed that SSRI-induced bruxism may actually manifest a form of SSRI-induced akathisia, involving centrally somatodendritic receptors on the cell bodies of raphé serotonergic neurons that project to the ventral tegmental area of the midbrain, and modulate the firing of the mesocortical tract from the ventral tegmental area to the prefrontal cortex for regulating masticatory muscle activity [60]. 

SSRI-induced bruxism [61] may lead to potential important microtrauma of the temporomandibular joint. This can be manifested pathologically in variety of ways, from thinning of the synovial temporomandibular joint disc that can lead to its perforation and result in bone-on-bone rubbing with crepitus or osteoarthrosis, to intrusion of the posterior molars and back-sliding of the front incisor and canine teeth to occlude on the posteriors teeth, with consequential posteriorization of the jaw and displacement of temporomandibular joint disc and increased risk for onset of an arthritic process at the temporomandibular joint. Animal studies confirm that depression induces bone loss, and therefore favor osteoporosis by means of a mechanism mediated by brain-to-bone sympathetic signaling. Whereas these effects can be alleviated by increasing central serotonin, the negative skeletal effects of the peripheral SSRI-induced increase in serotonin *per se* outweigh the osteoimmune benefits resulting from the enhanced central serotonin antidepressant and antisympathetic activity [62].

Therefore, these lines of evidence lead us to propose the hypothesis that the pathological condition of temporomandibular joint disorder and its *sequelae* are more prevalent than one might expect in patients with HIV/AIDS because of their related osteoimmunopathology. The probability that men and women with HIV/AIDS suffer from temporomandibular joint disorders with an incidence significantly greater than epidemiological statistics would presently suggest is clinically relevant. In many patients with disorders of the temporomandibular joint, the auriculotemporal nerve, a branch of the 2nd branch of the trigeminal cranial nerve (CV-II, maxillary branch), may be compressed, irritated, or otherwise damaged. This results in significant central and peripheral nerves via the gasserian ganglion and other central pathways, in precipitating a series of pathological manifestations reminiscent of a spectrum of neurological pathologies [63]. The need emerges, therefore, to construct clinical studies aimed at the clinical involvement of temporomandibular joint disorders in HIV/AIDS pathologies, and experimental studies directed at testing the putative involvement of the gasserian ganglion in neuroAIDS.

### 2.2. Immune Modulators of Bone Metabolism

Immune cells from the lymphoid and the myeloid lineages influence bone remodeling by exerting an impact on osteoclastogenesis. Activated T cells play a critical role in bone loss consequential to immunopathology associated with systemic viral infections, such as HIV, as well as chronic local bone and joint diseases, such as rheumatoid arthritis or inflammatory bowel disease. Similarly, and true to homeostatic regulation, T cells can be regulatory and even inhibitory of these bone forming or removing events [64–69]. 

Based on these observations, and on the present understanding of the immunopathology of HIV/AIDS, it is not unreasonable to expect, therefore, a complex and varied series of osteoimmunopathologies in asymptomatic HIV-seropositive patients, as well as in patients with fully developed manifestations of AIDS. For instance, the Immune Reconstitution Inflammatory Syndrome (IRIS) is often a considerable problem in the treatment of patients with HIV/AIDS. IRIS is characterized by highly elaborate and significant activation of both innate and adaptive immune responses with elevation of body fluid chemokines and cytokines, including common markers of inflammation such as C-reactive protein, interferon-inducible protein 10, and IFN-*γ*, and that together signify innate and adaptive immune activation [70]. 

IRIS-responding HIV-seropositive patients show higher frequencies of effector memory T cells positive for programmed death (PD)-1, a member of the extended CD28/CTLA-4 family of T-cell regulators, HLA-DR+, and Ki67+ in CD4+ T, and in regulatory T CD4+CD25+FoxP3+ cells, than IRIS-nonresponding otherwise matched HIV-seropositive patients. The PD-1+CD4+ T cells in the IRIS-responding patients express increased levels of LAG-3, CTLA-4, and ICOS, and are driven to a TH1/TH17 cytokine profile when stimulated *in vitro. *Plasma levels of IRIS-responding HIV-seropositive patients also show marked elevation in the TH1 cytokine IFN-*γ* as well as in IL-7, which together confirms that IRIS is predominantly CD4-mediated phenomenon directed at reconstituting efficacious activation of effector and regulatory T cells [71].

Of course, reconstitution of T-cell-mediated immunity is desirable in patients with HIV/AIDS, but overreconstitution of T-cell responses into a pronounced, unrestricted, and uncontrollable immune reconstitution inflammatory syndrome is less desirable. Here, we propose the hypothesis that, based upon our current understanding of osteoimmune interactions and of the incipient osteoimmunopathology in patients with HIV/AIDS, including increased bone fragility and decreased compact bone mineralization together, IRIS may actually dangerously increase the relative prevalence of osteoporosis in patients with HIV/AIDS.

We propose that one mechanism by which IRIS may induce osteoporosis in these patients is by means of increased TH17 activity. Lymphocytes expressing *γδ* T-cell receptors constitute an entire system of functionally specialized subsets that have been implicated in the regulation of immune responses, including responses to pathogens and allergens, and in tissue repair. *γδ* T cells represent a small subpopulation of T cells that, unlike *αβ* T cells, function more as cells of the innate immune system. *γδ* T cells are known to mediate the production of inflammatory cytokines, including interferon-*γ*, tumor necrosis factor-*α*, and interleukin (IL)-17 (i.e., TH17 cells) and thus enable the activation of other subsets of infiltrating effector cells. It is important to note, again perhaps in reference to neuroAIDS, that IL-17 and its receptor IL-17R have been implicated in the pathogenesis of immune-mediated CNS diseases [72].

Immune cells that are endowed with the ability to respond to challenges by means of IL-17 are in fact responsible for a wide variety of inflammatory and autoimmune disorders. Data demonstrate the presence of certain TH17 that are also capable of producing IFN*γ*, indicating a putatively overlapping subpopulation of TH17/TH1 cells [73, 74]. In brief, TH17 cells are characterized by surface expression of CCR6, IL-23R, IL-12Rb2, and CD161, expression of T-bet, the retinoic acid-related orphan receptor (ROR)*γτ*. As noted, they have the ability to produce IFN-*γ* and IL-17A in the presence of IL-12 and to arise from CD161+CD4+ precursors, which constitutively express ROR*γτ* and IL-23R, in response to the combined activity of IL-1*β* and IL-23. Whereas they generally are unresponsive to TGF-*β* for mediation of differentiation, they can favor their proliferation by inhibiting T-bet expression [74].

The IL-17 family of cytokines includes IL-17B, IL-17C, IL-17D, IL-17E (also called IL-25), and IL-17F. The primary function of IL-17-related cytokines is to modulate induction of many immune signaling molecules. The most notable role of IL-17 is its involvement in inducing and mediating proinflammatory responses and is commonly associated with allergic responses. IL-17 induces the production of many other cytokines (such as IL-6, G-CSF, GM-CSF, IL-1*β*, TGF-*β*, TNF-*α*), chemokines (including IL-8, growth related gene alpha, GRO-*α*, and MCP-1), and prostaglandins (e.g., PGE2) from many cell types (fibroblasts, endothelial cells, epithelial cells, keratinocytes, and macrophages). As a result, the IL-17 family has been linked to many immune/autoimmune-related diseases including osteoimmunopathologies such as rheumatoid arthritis and osteoporosis, which involve activation of osteoclastic bone resorption activity [31]. 

Each member of the IL-17 family has a distinct pattern of cellular expression. The expression of IL-17A and IL-17F is restricted to a small group of activated T cells and is upregulated during inflammation. IL-17B is expressed in several peripheral tissues and immune tissues. IL-17C is also found to be upregulated in inflammatory conditions, although in resting conditions it is low in abundance. IL-17D is highly expressed in the nervous system and in skeletal muscle and IL-17E is found at low levels in various peripheral tissues [75–77]. 

Although it has only limited homology to other cytokines, IL-17 exhibits pro-inflammatory properties similar to those of TNF*α*, particularly with respect to induction of other inflammatory effectors, including several bone pathologies, most notably rheumatoid arthritis [78]. Research has established that signal transduction pathways dependent on PI3K/Akt and NF-*κ*B are involved in bone-related pathology-mediated IL-17 possibly by modulating, in part at least, production of related pro-inflammatory cytokines (i.e., IL-6, IL-8) by synovial fibroblasts [79].

The IL-17 receptor family consists of five, broadly distributed receptors that present with individual ligand specificities. Within this family of receptors, IL-17R is the best described. IL-17R binds both IL-17A and IL-17F and is expressed in multiple tissues: vascular endothelial cells, peripheral T cells, B-cell lineages, fibroblast, lung, myelomonocytic cells, and marrow stromal cells. IL-17RB binds both IL-17B and IL-17E. Furthermore, it is expressed in the kidney, pancreas, liver, brain, and intestine. IL-17RC is expressed by the prostate, cartilage, kidney, liver, heart, and muscle tissues. The IL-17RC gene may undergo alternate splicing to produce a soluble receptor in addition to its cell membrane-bound form. In similar manner, the gene for IL-17RD may undergo alternative splicing to yield a soluble receptor. This feature may allow these receptors to inhibit the stimulatory effects of their yet-undefined ligands. The least described of these receptors, IL-17RE, is known to be expressed in the pancreas, brain, prostate, and bone [75–78].

In patients with HIV/AIDS, the role of TH17 cells and IL-17 have been under active investigation for some years [80], and the fine regulation of the TH17/Tregs ratio is now recognized as a critical predictor of HIV/AIDS pathogenesis [81], and Th17 populations are depleted in animal models of HIV infection and progression to AIDS [82]. 

### 2.3. Osteoporosis in Patients with HIV/AIDS

Taken together, the experimental evidence to date suggests that it is possible and even probable that patients with HIV/AIDS may be at increased risk for osteoporosis. Indeed, the clinical data appears to support this inference.

A recent modeling study defined and characterized the progression of low bone mineral density patients with HIV/AIDS. Predictive analyses based on linear regression and logistic polyatomic regression using in the polytomous model the categorical ranking of disease progression and severity were conducted on a longitudinal cohort sample of 671 patients. Clinical outcome measures consisted of repeated dual-energy X-ray absorptiometry scans, which yielded accurate estimates of bone mineral density, over 2-3 years. The prevalence of decreased bone mineral density in the sample was 47.5%, and the prevalence of osteoporosis was 23%. Clinically relevant progression to bone demineralization was observed in 28% of the patients, and 15.6% of the patients progressed to osteoporosis during study period. Statistically significant and clinically relevant predictors of these patterns of progression were age (odds Ratio (OR) : 1.07; CI_95_ = 1.05–1.08 (*P* < .0001), gender (male), OR : 2.23; CI_95_ = 1.77–2.8 (*P* < .0001), cachexia and wasting syndrome), OR : 1.14; CI^95^ = 1.11–1.17 (*P* < .0001), duration of treatment with protease inhibitor (PI), (OR : 1.18; CI_95_ = 1.12–1.24 (*P* < .0001), or with antiviral reverse transcriptase inhibitor (ART, tenofovir), (OR = 1.08; CI_95_ = 1.03–1.14 (*P* < .0019), and current PI treatment (OR : 1.64; CI_95_ = 1.35–2.04 (*P* < .0001) [83].

These findings were replicated in a cohort of 33 young men (mean age 38 ± 9) with HIV/AIDS (mean plasma HIV RNA: 5.0 ± 1.2 log10 copies/ml, and matched for body mass index (mean: 22.7 ± 3.3)). The cohort was followed longitudinally with a regular dual-energy X-ray absorptiometry of the lumbar spine, femoral neck, and total hip, and with clinical assessments for osteopenia and osteoporosis, defined by WHO criteria as T-scores between −1 and −2.5 and −2.5 or less, respectively. The data confirmed that the prevalence of low bone mineral density and elevated osteoporosis in young men with primary HIV infection was predicted by increasing age, HIV viremia, lower body mass index, and introduced an intriguing new variable: thyroid stimulating hormone levels [84].

In an earlier study, polytomous logistic regression also showed the significant predictive strength of age, homosexual transmission, low body mass index, and HIV plasma viral load for onset of bone abnormalities, defined in that study as decreased bone mineral density and increased osteoporosis and bone fragility, in men with HIV/AIDS. This study also showed older age, and low CD4 lymphocyte count nadirs were independently associated with osteoporosis in women with HIV/AIDS. However, the analysis failed to show the predictive strength of HAART for osteoporosis in these two groups, following adjustment and stratification for the considered anthropometric parameters [85]. That finding confirmed an earlier study with a Slovenian cohort of HIV/AIDS patients, in which the increased prevalence of osteoporosis was confirmed in a range similar to that reported by others (i.e., osteopenia: 47%, osteoporosis: 12%), but ART, PI, or HAART were not found to be significant predictors for these clinical outcomes [86] (Refer to pages 3, 12, and 13 for a complete perspective on PI/HAART/ART impact on osteoporosis. While there is evidence in support of both the association of PI/HAART/ART and osteopororis, as well as the opposing, this paper supports the clinical evidence for the direct link and intervention of PI/HAART/ART and osteopororis. However, for a full and thorough perspective of the case, articles in opposition of this connection are also cited.)

 It seems clear that the clinical data support the inference that based on our current understanding of osteoimmunology, patients with HIV/AIDS are expected to have increased prevalence of osteoimmunopathologies, including in particular osteoporosis. Increased methodological stringency and internal validity constraints will be required in future studies to establish beyond doubt the predictive role of ART, PI, and HAART for osteoporosis in patients with HIV/AIDS. 

## 3. Evidence-Based Practice for Osteoimmunopathology in HIV/AIDS: Osteoporosis

### 3.1. Treating Osteoporosis in Non-HIV/AIDS Patients

In the context of osteoporosis in patients who do not have HIV/AIDS, most treatments have some proven efficacy in reducing the risk of vertebral fractures. Overall however, the evidence is less convincing in terms of the prevention of nonvertebral fractures, in part because available randomized control trials (RCT's) typically yield *post hoc* subgroup analyses, rather than analyses based on the intent-to-treat. The intention-to-treat analysis aims to circumvent the effects of crossover and dropout, which alter the randomization to the treatment groups, and thus yield spurious data. In principle, the intention-to-treat analysis seeks to describe the potential effects of treatment policy rather than on the potential effects of a specific treatment, because it is a means of analyzing the outcome of a RCT based on the initial treatment intent, not on the treatment eventually administered. For the purposes of the intention-to-treat analysis, the entire sample that begins the treatment is considered to be part of the trial, whether they finish it or not [87]. In most recent development of this critical concept, the “modified intention to treat” (mITT) approach was proposed to provide some degree of internal control to sampling and sample allocation concerns consequential to the inclusion and the exclusions criteria imposed in a carefully crafted RCT [88].

A meta-analysis of eleven RCT's-Phase III was performed to compare the relative risks of nonvertebral antifracture efficacy for at least 3 years, confirmed by radiographs, of bisphosphonates, alendronate, and risedronate among several osteoporosis therapies in post-menopausal women. The research synthesis emphasized stringent assessment of the intention-to-treat sample. The analysis outcome established significant reductions in the relative risk of non-vertebral fracture for both alendronate (relative risk, RR = 0.86, CI_95_ : 0.76–0.97, *P* = .012) and risedronate (RR = 0.81, CI_95_ : 0.71–0.92, *P* = .001). Risedronate and strontium ranelate further evinced clinically relevant non-vertebral anti-fracture efficacy in the context of this research synthesis [89].

To further test and establish the strength of the clinical recommendations and the cost-effectiveness of selective estrogen receptor modulators, bisphosphonates, and parathyroid hormone for the prevention and in the treatment of osteoporosis, a systematic review was brought forward with the specific goal of contributing to the body of knowledge aimed at preventing or reducing of osteoporotic fractures in postmenopausal women. The process of research synthesis emphasized meta-analysis, rather than the complete traditional systematic review structure. Studies were included in a random effect model meta-analysis, if the outcome of fracture incidence was reported in terms of the number of patients suffering fractures. The clinical decision-making model was derived from the meta-analytical results and inference to estimate the cost-effectiveness of osteoporosis interventions by calculating the number of fractures that occurred, as a function of the costs associated with the osteoporotic fractures, and the quality-adjusted life years. As a reference control, the conditions of breast cancer and coronary heart disease were modeled by the same approach because certain interventions can affect the risk probabilities for these conditions. Using a sample size of ninety RCT's that met the inclusion criteria, a comparison of five interventions (alendronate, etidronate, risedronate, raloxifene, and teriparatide) to five reference control treatments (calcium, calcium plus vitamin D, calcitriol, hormone replacement therapy, and exercise), and to a no-treatment placebo controls was possible. The intervention costs of treating all osteoporotic women for 5 years with alendronate, etidronate, risedronate, or raloxifene was elevated, and the cost adjusted per quality-adjusted life years decreased dramatically with age. In fact, of the five tested interventions, only raloxifene appeared to reduce the risk of vertebral fracture in postmenopausal women, independently for low bone mineral density. However, the evidence indicated that none of the five interventions effectively reduces the risk of non-vertebral fracture in women, regardless of low bone mineral density, whereas they all led to substantial gains in quality-adjusted life-years, particularly among older women. The research synthesis also clearly evinced that the estimated costs varied widely across the interventions, age, and clinical profile (i.e., prior fracture) [90].

Research synthesis, followed by a Markov model of decision analytic techniques, was utilized to establish the comparative effectiveness and long-term costs and outcomes of five treatment and secondary prevention strategies for osteoporosis: placebo, “no intervention”, alendronate, etidronate, risedronate, and raloxifene, in postmenopausal (65+ year old) osteoporotic women without prior fracture. Probabilistic sensitivity analysis, which is directed at predicting how changes in a given model inputs can probabilistically influence the outputs with the purpose of determining “good practice” [91], was used to incorporate the impact of parameter uncertainty. In addition, deterministic sensitivity analysis, which differs from the former in that it seeks to predict how changes in a given model inputs can be determined by certain specified variables to influence the outputs, was used to compare alternative patient populations and modeling assumptions. Life years and Quality Adjusted Life Years were the outcomes of interest in the comparative effectiveness investigation, which established that risedronate was less effective than etidronate and alendronate. Alendronate and etidronate were as cost-effective alternatives for treating women with osteoporosis, although the model did not permit long-term projection [92].

A related research synthesis confirmed these findings, as it examined the cost-effectiveness of nonfracture side effects of osteoporosis treatments in women screened for osteoporosis at age 65, and treated osteoporotic subjects as recommended with hormone replacement therapy, raloxifene, or alendronate. This approach utilized the Markov model of osteoporosis disease progression to simulate costs and outcomes by means of calculations of incremental cost-effectiveness ratios of screen-and-treat strategies, relative to a no-screen and no-treat strategy. Disease progression parameters, as well as cost and quality-of-life parameters were outcomes of interest in this analysis. Results showed that screening and treatment with hormone replacement therapy act in concert to increase costs and to lower quality-adjusted life years, relative to the no-screen, no-treat strategy, except when the model includes the assumption of no fracture and thus no drug-related health effects. In conclusion, whereas raloxifene further increases costs and quality-adjusted life years, alendronate emerges from this research synthesis as the most cost-effective strategy relative to the no-screen, no-treat strategy: in fact, with a fairly good and acceptable cost-effectiveness ratio [93].

Fragility fractures cause significant morbidity and mortality. Effective osteoporosis treatment can reduce fracture incidence, but it is not known whether it is efficacious as well in reducing mortality. A research synthesis protocol was designed with the aim of determining whether effective osteoporosis treatment might be efficacious in reducing mortality. Two databases (i.e., Pubmed-MEDLINE, Cochrane Central Register of Trials), as well as some “gray” literature obtained from American Society for Bone and Mineral Research conference abstracts were consulted. Eligible RCT's were included if they established efficacy interventions other than estrogen and selective estrogen receptor modulators in preventing both vertebral and non-vertebral fractures over a study duration longer than 12 months. Studies (*n* = 8) of risedronate, strontium ranelate, zoledronic acid, and denosumab were included in the research synthesis. The consensus statement arising from the analysis was that treatment is efficacious in leading to a reduction in mortality (relative risk analysis I = 0.89, CI_95_ : 0.80–0.99, *P* = .036; relative risk analysis II = 0.90; CI_95_ : 0.81–1.0, *P* = .044), and that mortality reduction is independent from age or incidence of hip or other non-vertebral fracture. Treatment overall was actually all the more efficacious in reducing mortality among the older, frailer individuals with osteoporosis at high risk of fracture [94].

In a study aimed to review the pharmacology, pharmacokinetics, pharmacodynamics, safety, efficacy, and use of denosumab in osteoporosis, breast cancer, prostate cancer, and multiple myeloma, pertinent research papers and abstracts were identified through a complete search of the two specific databases, Pubmed-MEDLINE and International Pharmaceutical Abstracts, for the publications between 1966 and July 2009. Key search terms included denosumab, its former name AMG-162 and its trade name Prolia, as well as its recognized functional characteristics as a fully human monoclonal antibody that specifically targets the receptor activator of the nuclear factor-*κ*B ligand (RANKL) system. Indeed, the FDA has given priority review status to the RANKL inhibitor denosumab (Prolia) to reduce skeletal-related events in cancer. For this particular systematic review, all available human clinical studies were included, except for studies in rheumatoid arthritis and giant cell tumor of the bone. The research synthesis design evinced that in patients with osteoporosis, denosumab significantly reduced bone resorption and consequential fractures, in large part by increasing bone mineral density and reducing bone turnover markers of osteoclast-mediated function. Comparative effectiveness analysis determined that denosumab was at least as effective in reducing bone turnover markers as intravenous bisphosphonates in oncology patients. Efficacy analysis also established that patients with osteoporosis commonly reported side effects of denosumab as arthralgia, nasopharyngitis, back pain, and headache. By contrast, the most common adverse effects of denosumab intervention in patients with cancer were infection, often severe enough to require hospitalization, arthralgia, bone pain, and fatigue [95].

### 3.2. Patients with HIV/AIDS

Taken together, this research is particularly timely and critical to the HIV/AIDS pandemics. As noted above, HIV-seropositive adult men and women suffer from severely decreased bone mineral density and increased risk of osteoporosis-related fragility fractures. Data indicate that the prevalence of osteoporosis in HIV-infected individuals is more than three times greater compared with HIV-seronegative control subjects. As noted above, HIV+ patients treated with antiretroviral therapy (ART) or protease inhibitors (PI) evince an even higher prevalence of reduced bone mineral density and increased risk of osteoporosis, compared with their respective controls. This evidence was reviewed in a research synthesis design involving a random effect meta-analysis. The search of the available evidence was broad and included the MEDLINE, Pubmed, and EMBASE databases for peer-reviewed cross-sectional studies between January 1966 and November 2005. The PICO criteria included pooled odds ratios of reduced bone mineral density and increased osteoporosis, as outcomes: the following patient population groups: HIV-positive versus HIV-negative; the following comparative interventions: ART-treatment versus ART-naïve, and PI-treatment versus PI-untreated. The twenty studies that met all of the inclusion/exclusion criteria yielded 884 HIV-seropositive patients, with a prevalence of decreased bone density of 67% (pooled odds ratio [OR] : 6.4), of whom 15% manifested clinical signs of osteoporosis (pooled OR : 3.7), compared to control HIV-seronegative subjects (*n* = 654). Whereas studies did generally not correct for HIV/AIDS severity and treatment dose, regimen and duration, the data revealed that, compared with ART-naive patients (*n* = 202, 10 studies), ART-treated HIV-seropositive patients (*n* = 824) showed a 2.5-fold increased odds of decreased bone mineral density, and increased risk for osteoporosis (7 studies). Similarly, PI-treatment increased the overall risk for lower bone mineral density and greater risk of osteoporosis in HIV-seropositive patients [27] (Refer to pages 3, 10, and 12 for a complete perspective on PI/HAART/ART impact on osteoporosis. While there is evidence in support of both the association of PI/HAART/ART and osteoporosis, as well as the opposing, this paper supports the clinical evidence for the direct link and intervention of PI/HAART/ART and osteoporosis. However, for a full and thorough perspective of the case, articles in opposition of this connection are also cited.)

In a Cochrane systematic review, the effects of interventions aimed at increasing bone mineral density in HIV-infected adults were examined. Following a remarkable extensive search of the available evidence that included MEDLINE, EMBASE, LILACS, and The Cochrane Library as well as “gray literature such as Meeting Abstracts, AIDSTRIALS, ACTIS, Current Controlled Trials, National Institutes of Health Clinical Trials Registry, and CenterWatch, only randomized trials that compared pharmacological or nonpharmacological therapy with placebo, no treatment, or an alternative therapy, in seropositive men and women 18 years of age or older were included. The clinical outcome of interest was increasing bone mineral density. The extensive and focused nature of the systematic review yielded three RCT studies that reported examination of the role of alendronate in patients with HIV and osteopenia or osteoporosis. A meta-analysis was precluded because of excessive heterogeneity (*P* < .0001), which was attributable to marked differences in the study populations and interventions. Nonetheless, the sensitivity analysis showed that in two homogeneous studies (heterogeneity, *P* = .11), alendronate, calcium, and vitamin D markedly improved lumbar bone mineral density after one year, when compared with calcium and vitamin D alone (weighted mean difference: +2.65; CI_95_  =  0.80–4.51). Of note is the clinically relevant observation that the alendronate-supplemented group did not evidence fewer fragility fractures (relative risk [RR] : 1.28; CI_95_ = 0.20–8.21), or osteoporosis symptomatology (RR : 0.50; CI_95_ = 0.24–1.01), and, overall, adverse occurrence of clinical outcomes was not significantly different between groups (RR : 1.28; CI_95_ = 0.20–8.21). One RTC, markedly heterogeneous with the others, demonstrated that patients with AIDS wasting showed clinically important improvement in lumbar bone mineral density following three months of testosterone enanthate treatment, compared to placebo, (weighted mean difference: +3.70; CI_95_ = 0.48–6.92), whereas progressive resistance training failed to improve this outcome in the patients. Neither the testosterone nor the resistance-training group suffered adverse effects. Taken together, and as briefly outlined above, the best available evidence that has emerged from this systematic review confirmed that bisphosphonate therapy in HIV-seropositive adults, and testosterone in patients with AIDS wasting syndrome appears to be both safe and possibly effective to improve bone mineral density. Clearly, these conclusions are preliminary due to the limited number of pertinent and homogeneous studies included in this analysis [25]. Evidently,, the question remains unanswered as to the clinical efficacy and effectiveness of alternate interventions, such as estrogen, testosterone, calcitonin, and teriparatide, which are less studied in HIV-seropositive patients.

### 3.3. Conclusion: Toward Comparative Effectiveness-Efficacy Research and Analysis For Practice (CEERAP): A New Frontier

Research to date indicates that osteoporosis is one clinically relevant pathology in patients with HIV/AIDS, and that our treatment armamentarium proposes several medications to treat this condition, depending on age and gender of the patient, severity of the HIV/AIDS pathology, and PI, ART, or HAART course of treatment. However, the problem remains as to what degree can the presently available lines of evidence be taken as concerted and confluent, rather than contrasting and contradictory. In several instances, research seeks to demonstrate that a given treatment modality “works” in arresting the bone resorption process, that is, the research evidence proffers and supports the efficacy of the intervention. Alternatively, research evidence may seek to emphasize cost-effectiveness and benefit-risk ratio effectiveness.

Thus, the body of studies outlined above can be loosely classified as either seeking to compare the efficacy of the treatment intervention for immediate utility in practice, or to compare the effectiveness of the modality in terms of potential costs incurred, benefits received, with possible exposure to risks (e.g., treatment of osteoporosis with bisphosphonates may be efficacious, that is, “it works”, in arresting bone resorption, but its effectiveness in terms of the increased risk of osteonecrosis, particularly of the mandibular and to some lesser extent of the maxillary bones, may be prohibitive.

The search for the best available evidence for efficacious and effective treatment intervention is a concerted effort by the research community to translate statistical significance data obtained in group comparisons yielded by RCT's and observational studies into clinically relevant consensus revisions of practice guidelines [96]. This is the fundamental outcome obtained by research synthesis, that is, the process of obtaining the entire body of available research evidence that pertains to a given patient population (here of interest: patients with HIV/AIDS and clinical signs of osteoporosis), treatment interventions that the clinician might consider, and thus of which a comparison is necessary in terms of efficacy or effectiveness, for a given clinical outcome of interest, within a set timeframe and a given clinical and socioenvironmental setting. Hence, the acronym of the research question of the research synthesis process is posed as P-I-C-O-T-S [31].

As in any cogently planned and conducted research, the scientific process, in this case the PICOTS question, can be simply the fundamental hypothesis of the study. Therefore, for the research synthesis to yield the best available evidence is a hypothesis-driven process by which the identified available body of evidence that pertains to the PICOTS question is analyzed for the level and the quality of the evidence through quantifiable means. This approach permits statistical inferences based upon acceptable sampling, heterogeneity analysis, and meta-analysis In this manner, research synthesis leads to the articulation of an analysis of the consensus of the best available evidence, which serves the clinician for clinically relevant efficacy and effectiveness decisions.

In brief, the process outlined in the preceding paragraphs is a hypothesis-driven systematic search for the consensus of the best available evidence for treatment. It yields a systematic research synthesis of comparative effectiveness and of comparative efficacy, the cogent analysis of which converges in patient-centered and patient-optimized evidence-based practice. It often involves the synthesis of primary research (RCT's, observational studies) to yield technical research synthesis reports, which are referred to as “systematic reviews”. Increasingly, research synthesis protocols involve the systematic evaluation of multiple existing systematic reviews, a process which must not remain purely technical but must strive to establish the clinical relevance of the consensus evidence: hence, the term “clinically relevant complex systematic review” [97].

Systematic reviews and clinically relevant complex systematic reviews are used in research and analysis comparing either the effectiveness or the efficacy of treatment intervention. However, the “real world” of patient care and practice demands that comparative effectiveness and comparative efficacy research not be distinct, but in fact remain conjoined for the ultimate administration of the best treatment intervention in terms of what works, and of what is most cost-effective, beneficial, and poses the least amount of risk to the patient. Research synthesis in health care must focus on quantifying the quality of the evidence and the strength of the clinical recommendations, in a manner to our expansion of the GRADE assessment tool (Phi et al., manuscript in preparation). Hence, we propose that the new frontier in the domain of research synthesis in health care will be the integration of these two traditionally distinct avenues into a novel perspective, which could be termed Comparative Effectiveness-Efficacy Research & Analysis for Practice (CEERAP).

In conclusion, it is clear that HIV/AIDS has posed a challenge to fundamental and clinical research for decades. Surely, this is so in large part because the term actually implies to at least three distinct epidemiologies: in the Western societies, we note a substantial difference between patients with HIV/AIDS in the population of multidrug users and patients who have contracted HIV/AIDS following unsafe sexual practices. There may be some degree of overlap between these groups in some instances, but mostly they represent two distinct patient populations from the socioeconomical viewpoint. In emerging societies and in the developing world, the HIV/AIDS epidemic is different altogether, as it affects the pediatric population in greater number, it is more prominent, and causes considerably more deaths than in the West. In the heart of Africa, entire villages may be affected, a large majority of the population of certain countries (e.g., Swaziland) may be affected with osteoimmune and other HIV/AIDS pathologies, which calls for an even greater urgency for a globalization of CEERAP (g-CEERAP).

The success of our intervention efforts to combat and defeat HIV/AIDS worldwide in the next decade will rest on g-CEERAP, we contend, along three principal domains:

- identification of the best available evidence for effectiveness and efficacy of treatment intervention for osteoporosis as a pathology of HIV/AIDS,
- dissemination in order to increase health literacy of the patient and caregiver populations, as well as the health care providers who may not be familiar with CEER analysis for practice,
- worldwide access via 2G or 3G to paperless CEERAP recommendations for full coverage of human information technology.
